# Factors Associated with Knowledge, Attitudes, and Prevention towards HIV/AIDS among Adults 15–49 Years in Mizoram, North East India: A Cross-Sectional Study

**DOI:** 10.3390/ijerph19010440

**Published:** 2021-12-31

**Authors:** Lucy Ngaihbanglovi Pachuau, Caterina Tannous, Kingsley Emwinyore Agho

**Affiliations:** 1 Campbelltown Campus, School of Health Science, Western Sydney University, Penrith, NSW 2560, Australia; C.Tannous@westernsydney.edu.au; 2 Campbelltown Campus, Translational Health Research Institute (THRI), Western Sydney University, Penrith, NSW 2571, Australia; 3 African Vision Research Institute (AVRI), University of KwaZulu-Natal, Durban 4041, South Africa

**Keywords:** human immuno-deficiency virus, HIV, India, knowledge, attitude, acquired immune deficiency syndrome, AIDS, HIV infection, HIV transmission

## Abstract

Despite a campaign of effective educational interventions targeting knowledge, attitudes, and prevention, Human Immunodeficiency-Virus/Acquired Immune Deficiency Syndrome (HIV/AIDS) continues to be a significant public health issue in India, with Mizoram reporting the highest HIV/AIDS cases in 2018–2019. In this study, we extracted Mizoram state from the National Family Health Survey Fourth Series (NFHS-4) 2015–2016 datasets and investigated factors associated with respondents’ knowledge, attitudes, and prevention towards HIV/AIDS. The sample included 3555 adults aged 15–49 years residing in Mizoram, North-east India. Respondents who reported having ever heard of HIV/AIDS was 98%. Multivariate analysis indicated that the probability of having inadequate knowledge of HIV/AIDS was higher among those with no schooling, who were illiterate, of non-Christian faiths, belonging to backward tribes or caste, from poor households, and those who lived in rural areas, not exposed to media. The odds of mother-to-child transmission (PMTCT) of HIV/AIDS transmission was high among females (AOR = 3.12, 95% CI 2.34–4.16), respondents aged 35–39 years (AOR = 1.74, 95% CI 1.05–2.87) and those belonging to other backward class. The HIV/AIDS knowledge of respondents was found to be encouraging as the majority (98%) were considered to have a good level of understanding of the condition. An educational intervention to reduce the number of adults 15–49 years infected with HIV/AIDS in Mizoram should target those from low socioeconomic groups, those from non-Christian religions, and those from other backward classes.

## 1. Introduction

Human Immunodeficiency Virus (HIV) continues to be a major global public health issue, with thousands of new HIV infections every year, a disease that has claimed almost 33 million lives so far [1]. In 2019, 38.0 million people globally lived with HIV, and there were 1.7 million new HIV infections and 690,000 Acquired Immuno-Deficiency Syndrome (AIDS) related deaths alone in the same year [2]. India has the third-largest HIV epidemic globally and reported its first cases of HIV in 1986 among female sex workers in Chennai, South India, followed by a rapid increase in many other States and peaked in 1997. In 2019, HIV prevalence among adults (aged 15–49) was estimated to be 0.22%; this figure may seem small, but it equates to 2.3 million people living with HIV (PLHIV) in India.

In 2019, around 69.22 thousand new infections and AIDS-related deaths were estimated at 4.43 per 100,000 population [3]. However, India has made significant progress in tackling its epidemic, with a 37% decline in new HIV infections since 2010 [3]. National AIDS Control Organization (NACO) is the body responsible for formulating policy and implementing programs to prevent and control the HIV epidemic in India. The National AIDS Control Programme (NACP) was also established in order to control the spread of HIV/AIDS by raising awareness and promoting the use of preventive measures in the general population.

The North-eastern state of Mizoram has the second-highest AIDS mortality in India at 23.34 per 100,000 population. While the new HIV infection incidence is declining in the country and other States, the State of Mizoram has seen an 18% increase in incidence from 2010 to 2017 [4]. There were an estimated 20.05 per 1000 people living with HIV in Mizoram and an estimated 1.18 HIV incidence per 1000 of the uninfected population in 2019 [3]. Mizoram State AIDS Control Society (MSACS) reported an average of 9 cases of HIV detected per day in Mizoram [5], an alarming rate in a state with a population of only 1.1 million. The HIV status in Mizoram has continued to remain stably high between 2010 and 2019 [3].

In 1992, the Government of Mizoram established the State AIDS Cell, and Mizoram State AIDS Control Society (MSACS) came into existence in 1998 under Health and Family Welfare. Since then, MSACS has promoted and implemented education and awareness programs to prevent and control HIV/AIDS in Mizoram. Despite the efforts made by MSACS in controlling the spread of HIV, the number of new infections is increasing; this causes an increased concern regarding the level of awareness, beliefs, and practices in HIV/AIDS in the general population.

A recent study in India has indicated the effectiveness of education on improving the knowledge and attitudes of young women toward HIV [6]. Several studies have reported social misconceptions about HIV/AIDS that has led to stigmatization and discrimination of PLHIV, and this emphasizes the need to develop a high level of public awareness that facilitates the need for the development of intervention programs to fight stigma and ensure a safer environment for those that are affected [7,8,9]. In the past, few studies in India have accessed the knowledge, attitude, beliefs, and practices in issues related to HIV/AIDS. The National Integrated Biological and Behavioural Surveillance (IBBS) 2014–2015 was the first nationwide community-based surveillance among high-risk groups (HRG) and bridge population that collected information on key parameters including knowledge indicators related to HIV prevention, STI, condom use, HIV/AIDS services, as well as experiences of stigma and discrimination towards people living with HIV/AIDS [10]. Another study in India has reported the urgent need to facilitate greater care and acceptance of people living with HIV/AIDS by raising the community’s level of knowledge about HIV and its modes of transmission [11].

There have been no studies that have assessed people’s knowledge, attitudes, and beliefs towards HIV/AIDS in Mizoram. A study in India has reported the urgent need for and efforts toward making people more caring and accepting towards PLHIV. This can be mainly achieved by raising knowledge about HIV and its modes of transmission [11]. In the absence of any curable treatment for HIV/AIDS, prevention of transmission through correct knowledge, positive attitude, safe behavior, and practices are the only practical way to contain HIV/AIDS [8]. Mizoram state in India still has the highest HIV prevalence and incidence rate [5]. Hence, there is an urgent need to analyze and determine the knowledge, attitude, and beliefs regarding HIV/AIDS among the adult population (15–49 years). This study was done by analyzing the National Family Health Survey IV (NFHS-4) findings in 2015–2016 on the current levels of HIV/AIDS knowledge, attitudes, and beliefs of the adult population in Mizoram, India. The findings of this study would aid in the establishment of a basis for further research in this field and help programs and policymakers modify their approach toward HIV/AIDS.

## 2. Materials and Methods

### 2.1. Data Sources

The study used data from the 2015–2016 National Family Health Survey Fourth Series (NFHS-4) [12]. NFHS is a large-scale, multi-round survey conducted in representative households throughout each state of India. NFHS-4 survey was conducted under the Ministry of Health and Family Welfare (MoFHW) supervision, Government of India, and technical facilitation was guided by the United States Inner City Fund (ICF) International Maryland. A two-stage sampling design was administered in rural areas as well as urban areas. In rural areas, the sample was selected through a two-stage design with villages as Primary Sampling Units (PSUs) based on the probability proportional to the size of the sample. The second stage involved selecting 22 households from each PSU randomly with systematic sampling. The two-stage sample design was also used in urban areas with Census Enumeration Blocks (CEB) chosen at the first stage and randomly selected 22 households in each CEB at the second stage [13]. The fieldwork in Mizoram was conducted in 8 districts by Research Development Initiative and gathered information from 11,397 households, 12,279 women aged 15–49 years, and 1749 men aged 15–54 years.

Four survey questionnaires were administered that collected information on household data and interviewed eligible women and men and biomarkers. Two versions of questionnaires were used for women in NFHS-4. The first version (district module) questionnaire did not include questions on sexual behavior and HIV/AIDS. This version of the questionnaire was fielded in the entire sample of NFHS-4 households. In the second version of the questionnaire (state module), four additional topics, including sexual behavior and HIV/AIDS, were included. This second version was fielded from a 15 percent subsample of NFHS-4 households designated to provide information only at the state and national levels. Men had only one questionnaire [12,13]. Information on socio-demographic variables (such as age, gender, religion, caste, education, wealth index, and marital status) and knowledge, attitude, and sexual practices in HIV/AIDS were collected from a sample of men and women aged 15–49 years. In addition, the NFHS-4 sample was designed to provide general population estimates of HIV prevalence for women and men for 11 groups of states/union territories in which we extracted Mizoram state for our data analysis. Hence, the final sample analyzed for this study consisted of 1973 women and 1625 men.

### 2.2. Potential Confounding Factors

The analyses were guided by previous studies [7,14,15], especially from low-and middle-income countries, which played a role in the potential confounding factors selected for the study and were classified into three levels: individual-, household- and community-level factors. Individual-level factors were respondents’ gender, working status, education, age, religion, and marital status. Household-level factors were household wealth index, respondents’ literacy, and amount of regular media exposure (exposure to either radio, television, newspapers, or magazines at least once a week), while community-level factors included type of residence, migration status, and caste/tribe. A similar study was done in Ecuador [16] also found education level and household wealth as potential confounding factors in their study.

A wealth index was constructed from the data collected through the household questionnaire using the method recommended by the World Bank Poverty Network and United Nations Children’s Fund [17]. The principal components statistical procedure was used to determine the weights for the wealth index based on information collected from about 22 household assets and facilities in order to estimate the household wealth index factor score. The household wealth index factor score was divided into three categories, and each household was assigned to one of these wealth index categories. The lowest quintile, the bottom 40% of the households, were referred to as the poorest households, the next 40% as middle households, and the highest quintile top 20% as the richest households [18]. To avoid correlation within the individual-, household- and community-level factors, we tested and reported any collinearity in the final model.

### 2.3. Outcome Variables

The four outcomes that were analyzed in this study can be broadly classified under the following headings.

#### 2.3.1. Knowledge of HIV/AIDS

This included questions on those who have heard of HIV or AIDS.

#### 2.3.2. Attitude towards HIV/AIDS

The questions included combining those that says ‘YES’ to all the four indicators “willingness to care for a relative with HIV/AIDS in own home”, “would they buy vegetables from a shopkeeper or vendor who has HIV/AIDS”, “female teacher who has HIV/AIDS but is not sick should be allowed to continue teaching”, “would not want to keep a secret that a family member got infected with HIV/AIDS”.

#### 2.3.3. Prevention towards HIV/AIDS

These included questions on reducing the risk of getting HIV/AIDS combined with those that said ‘YES’ to (a) have 1 sex partner only, who has no other partners, and (b) always use condoms and 1 sex partner only, who has no other partners.

#### 2.3.4. Prevention of Mother-to-Child Transmission (PMTCT)

These included questions on knowledge of prevention of HIV/AIDS from mother-to-child transmission (PMTCT) and that HIV/AIDS transmission from mother to her baby can occur (a) during pregnancy, (b) during delivery and, (c) by breastfeeding.

### 2.4. Statistical Analysis

All analyses were performed using “svy” commands in STATA v14.1 (Stata Corporation, College Station, TX, USA), and the binary form of the four outcome variables was noted as 1 for “Yes” and 0 for “No”. Preliminary analyses involved frequency tabulations of all selected characteristics, followed by the Taylor series linearization method [19] used in the surveys when estimating 95% confidence intervals around prevalence estimates. Cross tabulations were generated to describe the frequencies and confidence intervals of the 4 outcome variables which are indicators across explanatory variables, and the statistical significances were tested using a chi-squared test.

For selected indicator variables, the unadjusted factors were tested by odds ratios (OR) using univariate analyses that adjusted for cluster, and survey weights were presented as unadjusted OR (95% CI) for all explanatory variables. Then, multiple logistic regression analyses that adjusted for cluster and surveyed weights in a manual process stepwise backward regression model [20,21] were used to identify those factors associated with knowledge, attitude, and prevention towards HIV/AIDS among adults 15–49 years in Mizoram, Northeast India and then presented as adjusted OR (95% CI) for the variables retained in the final step.

## 3. Results

### 3.1. Characteristics of the Sample

Summarized in Table 1 is the individual, household, and community-level characteristics of HIV/AIDs among adult males and females aged 15–49 years in Mizoram, the majority of the respondents lived in rural areas (61.9%). More than half (55%) of the respondents were employed in the last 12 months, and 84.6% had a secondary or higher level of education. Of the total respondents, more than half (55.3%) were female. Most of the respondents were currently married (51.3%), and 97.6% belonged to the scheduled tribe. The proportion of individuals who could read a sentence was 91.7%, about one-third (26.8%) were from poor households, and 95.1% belonged to the Christianity faith.

The 95% confidence intervals of 4 outcome variables across all explanatory variables were calculated. Results indicated that low levels of education, other backward class, poor household wealth index, belonging to a religion other than Christianity, not having regular exposure to media, being illiterate, and living in rural areas commonly reported significantly lower knowledge of HIV/AIDS, prevention towards HIV/AIDS, PMTCT and attitude towards HIV/AIDS (Supplementary Table S1 for details).

### 3.2. Knowledge, Attitude and, Prevention of HIV/AIDs: Proportion of ‘Yes’ Respondents

Of the sample of 3555 adults aged between 15 and 49 years from Mizoram, the proportion of individuals who had ever heard of HIV/AIDS was 98%. The proportion of individuals who reported always using condoms during sex was 97.4%; 37.4 reported that HIV/AIDS would be kept secret in the family, and 84.2% reported knowing that HIV can be transmitted from mother to child. The proportions of individuals who expressed positive or accepting attitudes toward people with HIV/AIDS; such as agreeing that an HIV positive female teacher who was not sick should be allowed to continue teaching, or that they would buy vegetables from an HIV positive vendor, and would care for a relative with HIV/AIDS were 95.5%, 91.4%, and 97.1%, respectively (Table 2).

Table 3 illustrates that the following factors were significantly associated with inadequate knowledge of HIV/AIDS: those who were illiterate (*p* < 0.001), those from a non-Christian faith (*p* < 0.001), with no regular media exposure (*p* = 0.006), and those who lived in rural areas. Knowledge on the prevention of HIV/AIDS was lower among those of non-Christian faith (*p* < 0.001), no regular media exposure (*p* = 0.023), and those from other backward class (*p* = 0.03). Negative attitude towards HIV/AIDS was significant among those from a rich household wealth index (*p* = 0.02), those exposed to media (*p* = 0.028), and those from other backward class (*p* = 0.002).

Only factors identified as significant were included in a multivariate analysis and are reported in Table 4. Several factors were associated with having inadequate knowledge of HIV/AIDS; respondents who were of non-Christian faith (AOR = 0.28, 95% CI: 0.14, 0.55), having no education versus having primary education (AOR = 3.05, 95% CI:1.46, 6.37) or secondary education (AOR = 0.21, 95% CI:0.06, 0.73), who were illiterate (AOR = 0.07, 95% CI: 0.01, 0.31), those who lived in rural areas and who had no regular media exposure. The probability of having poor knowledge on prevention of HIV/AIDS was higher among those of non-Christian faith (AOR = 0.28, 95% CI: 0.14, 0.55), respondents from other backward classes (AOR = 8.60, 95% CI: 1.72, 43.02) and those who had no regular media exposure. The odds of knowledge of prevention of mother-to-child transmission (PMTCT) of HIV/AIDS was high among females (AOR = 3.12, 95% CI: 2.34, 4.16), respondents between the age of 35–39 years (AOR:1.74, 95% CI: 1.05, 2.87). Negative attitudes towards HIV/AIDS were significant among those of other backward classes (AOR:0.18, 95% CI: 0.07, 0.44).

## 4. Discussion

This study examined the knowledge, attitude, and preventive behaviors towards HIV/AIDS among adults in Mizoram. It found that those who lived in rural areas, from poor households, who were illiterate, with no schooling, from a non-Christian faith, belonging to other backward classes, and who had no media exposure to be factors associated with HIV/AIDS for this population group. This was the first study that sought to understand HIV/AIDS and its associated factors among adults in Mizoram. This study has revealed their shortcomings towards HIV/AIDS literacy and has identified areas for information, education, and communication interventions that will modify the approach toward addressing HIV/AIDS in Mizoram.

This study showed that the adult population in Mizoram had good overall HIV/AIDS knowledge, but that there were significant gaps among those who lived in rural areas, from poor households, who were illiterate, with no schooling, from a non-Christian faith, belonging to other backward class, and those who had no exposure to media. A similar study in Yemen [7] also found that rural dwellers and those with no schooling had significantly poorer knowledge of HIV/AIDS. Similarly, a study in India [22] found HIV transmission and prevention knowledge was low among women and rural residents. In contrast, a study in Karnataka, India [23] found no significant difference in the knowledge scores between urban and rural dwellers. This was believed to be due to an overall increase in awareness about HIV/AIDS through educational campaigns introduced by the government, non-governmental, international, and community-based organizations.

The study findings showed that respondents from other backward classes had low knowledge and prevention toward HIV/AIDS. The other backward classes of Mizoram are remotely isolated with limited access to media and health resources and have a high level of poverty and low level of education. A study among the rural tribal communities of Southern India [24] revealed that knowledge and awareness about HIV/AIDS were very low in this community. This was attributed to the high level of poverty, inadequate health resources, low literacy level, and minimal access to information. Our study showed the importance and the need to disseminate HIV/AIDS-related knowledge and awareness among socially and economically disadvantaged communities and those living in rural areas of Mizoram. Awareness and education programs geared specifically towards these communities delivered through trained local health workers are vital to curtail the increasing threat of HIV/AIDS in Mizoram.

The findings from our study showed that respondents who did not have Christian faith had lower knowledge and prevention of HIV/AIDS. This is similar to a study done in Ghana [25] and Lebanon [26] that revealed that respondents from the Christian faith demonstrated higher knowledge of HIV/AIDS than other religions. The majority (87%) of the Mizoram population are Christian, and the culture is largely influenced by Christianity. The church has played vital roles in the development and promotion of the socioeconomic and political status of the state [27]. However, a recent community needs assessment on HIV/AIDS [28] in Mizoram showed that the church did not take part in providing sex education or information on the prevention of HIV/AIDS. The church cannot and does not promote condom use but stigmatizes those who need it [28]. Church leaders in Mizoram agreed that they have responsibilities towards HIV prevention but were restricted by church doctrines [29]. Despite the church’s lack of contribution to HIV awareness and prevention in the community, the knowledge of HIV among Christians was higher. Further studies need to look into this and find ways to involve the church in disseminating HIV/AIDS-related knowledge and awareness in the community without compromising the church doctrines.

The majority of respondents were willing to care for HIV-infected relatives and would buy vegetables from an HIV-positive vendor. This revealed that the majority of the respondents had a positive attitude towards people living with HIV. However, 37.4% of the respondents reported that HIV/AIDS should be kept a secret in the family. The reports are similar to other studies done in India [11,30]. In India, social stigmatization and discrimination of people living with HIV are highly prevalent; lack of adequate knowledge, negative perception, variations in the socio-cultural taboos might underlie this negative attitude [31]. Families that have a member with HIV may fear isolation and ostracism within the community [32]. Consequently, they may try to conceal an HIV diagnosis which may cause considerable stress and depression within the family [32]. Intensive education and behavior change for people living with HIV/AIDS, their family members, and other stakeholders are required to curb HIV/AIDS and its related stigma and discrimination [33].

Regarding sexual behavior, the majority of respondents in the study reported always using condoms during sex and having only one sex partner. This could be attributed to the efforts made by the government and social organizations in propagating the use of condoms to prevent HIV through media, posters, and pamphlets. However, other studies [34,35] have pointed out that surveys of sexual behavior are always subjected to presentation bias, that is, participants giving socially desirable answers rather than the truth. The possibility of bias in the present study could not be ruled out.

### 4.1. Study Limitations and Strength 

Our study has several limitations. Data for our analysis were generated by self-report and thus may be subjected to presentation bias with respondents giving socially desirable answers rather than the truth. In addition, because our study was population-based, it did not differentiate high-risk groups such as female sex workers, injecting drug users, and men who have sex with men. Despite these limitations, this study has a very strong internal validity because this is a large sample-size population-based study, and the findings could help design and improve HIV/AIDS education and prevention programs in Mizoram.

### 4.2. Recommendations

HIV/AIDS education and awareness programs need to be disseminated among socially and economically disadvantaged communities, rural communities, and those from non-Christian faiths. This study showed that those who were illiterate had inadequate knowledge and understanding of HIV/AIDS. However, posters and pamphlets may not produce the desired knowledge regarding HIV among these groups of people. Mass media has been one of the important strategies in disseminating HIV/AIDS knowledge and promotion of HIV testing [36]. A study done in India [23] found that television played an important role in influencing HIV/AIDS knowledge and attitudes. In this study, 98.2% of respondents had regular media exposure. Increased utilization of mass media such as radio and television will be useful and beneficial in HIV/AIDS prevention, stigma reduction, and risk-reduction behaviors among the general population in rural and urban communities of Mizoram.

The church plays an influential role in the communities and reaches every corner of Mizoram [28]. HIV/AIDS awareness and education campaigns through the church would be beneficial in the prevention of HIV. Content of these education programs in a way that will not hurt or compromise the church doctrine and sentiments need to be developed.

## 5. Conclusions

This study demonstrated that HIV/AIDS knowledge level is encouraging in the study population, with the majority of the respondents reported to had heard of HIV/AIDS and understood the route of transmission. Nevertheless, HIV/AIDS prevention and education intervention need to be targeted toward those from low socioeconomic groups, from non- Christian religions, and those from other backward classes. The inadequate knowledge exhibited by those from rural communities warrants urgent and appropriate interventions and education on HIV/AIDS and its transmission, prevention, and reduce stigmatization and discrimination. The majority of the respondents reported having regular media exposure, increased utilization of mass media such as television and radio are recommended to disseminate HIV/AIDS awareness and education so that it reaches all strata of educational levels, socioeconomic groups, and religions within Mizoram.

## Figures and Tables

**Table 1:** Individual, household and, community-level characteristics of HIV/AIDs among adults aged 15–49 years in Mizoram, India 2015–2016 (*n* = 3555).

Characteristic	*n* (Weighted)	%	*n* (Unweighted)	%
***Individual-level factors***				
**Gender**				
Male	1588	44.7	1617	45.5
Female	1967	55.3	1938	54.5
**Respondents age in years**				
15–19	608	17.1	602	16.9
20–24	542	15.2	501	14.1
25–29	559	15.7	608	17.1
30–34	556	15.7	555	15.6
35–39	515	14.5	541	15.2
40–44	301	8.5	292	8.2
45–49	474	13.3	456	12.8
**Marital status**				
Never married	1425	40.1	1319	37.1
Currently married	1825	51.3	1983	55.8
Formerly married	305	8.6	253	7.1
**Educational status**				
No education	137	3.8	147	4.1
Primary	410	11.5	500	14.1
Secondary or more	3008	84.6	2908	81.8
**Working status in the last 12 months**				
Not working	1585	44.7	1473	41.6
Working	1964	55.3	2065	58.4
**Religion**				
Christianity	3382	95.1	3408	95.9
Others ^$^	173	4.9	147	4.1
***Household-level factors***				
**Household wealth index**				
Poor	952	26.8	1173	33.0
Middle	1053	29.6	1178	33.1
Rich	1550	43.6	1204	33.9
**Regular media exposure**				
No	65	1.8	65	1.8
Yes	3470	98.2	3490	98.2
**Literacy**				
Can read whole sentence	3260	91.7	3178	89.4
Cannot read whole sentence ^#^	295	8.3	377	10.6
***Community-level factors***				
**Area of residence**				
Rural	1354	38.1	1716	48.3
Urban	2201	61.9	1839	51.7
**Migration status**				
Yes	425	12.0	360	10.1
No	3130	88.1	3195	89.9
**Caste/Tribe**				
Scheduled Tribe	3471	97.6	3469	97.6
Other Backward Class	84	2.4	86	2.4

^$^ Hindu, Muslim, Buddhist/neo-Buddhist and other religion; ^#^ cannot read at all, can read only parts of a sentence.

**Table 2:** Responding ‘yes’ to the question about knowledge, attitude, and prevention towards HIV/AIDS.

Indicator	Female		Male			Both	
	*n*	% (95%CI)	*n*	% (95%CI)		*n*	% (95%CI)
Knowledge of HIV/AIDS (heard of HIV, *n* = 3555)							
Yes	1920	97.6 (96.8, 98.20)	1565	98.52 (97.71, 99)		3485	98.04 (97.48, 98.47)
Always use condoms during sex (*n* = 3377)							
Yes	1794	97.11 (95.87, 97.98)	1494	97.69 (95.77, 98.75)		3288	97.37 (96.37, 98.1)
Have one sex partner only, who has no other partners (*n* = 3352)							
Yes	1720	93.59 (91.69, 95)	1397	92.27 (89.27, 94.48)		3117	92.99 (91.36, 94.34)
Prevention towards HIV/AIDS (Always use a condom and one sex partner only, who has no other partners, *n* = 3271)							
Yes	1628	91.18 (89.05, 92.93)	1339	90.16 (86.83, 92.71)		2967	90.72 (88.8, 92.28)
HIV/AIDS can be transmitted from mother to her baby during pregnancy (*n* = 3054)							
Yes	1511	88.7 (85.9, 90.9)	1059	78.5 (74.0, 82.4)	2570	84.2 (81.7, 92.28)	
HIV/AIDS Can be transmitted from mother to her baby during delivery (*n* = 2553)							
Yes	1259	85.6 (82.2, 88.4)	796	73.6 (68.5, 78.1)	2055	80.5 (77.6, 83.1)	
HIV/AIDS can be transmitted from mother to her baby during breastfeeding (*n* = 2894)							
Yes	1513	93.1 (91.1, 94.8)	1014	79.9 (75.6, 83.6)	2527	87.3 (85.1, 89.2)	
PMTCT (Who know that HIV can be transmitted from mother to child, *n* = 2541)							
Yes	1072	74.3 (70.4, 77.9)	528	48.1 (43.1, 53.1)		1600	62.9 (59.6, 66.2)
Willing to care for a relative (*n* = 3382)							
Yes	1802	96.73 (95.26, 77.76)	1481	97.47 (95.5, 98.59)		3283	97.07 (95.96, 97.88)
HIV in family to remain secret (*n* = 3026)							
Yes	688	39.13 (35.24, 43.15)	442	34.87 (30.25, 39.78)		1130	37.34 (34.35, 40.43)
HIV female teacher not sick to continue teaching (*n* = 3382)							
Yes	1795	94.75 (92.74, 96.22)	1433	96.36 (94.58, 97.57)		3228	95.45 (94.14, 96.48)
Would buy vegetables from HIV positive vendor (*n* = 3328)							
Yes	1690	92.44 (90.4, 94.07)	1353	90.2 (87.38, 92.45)		3043	91.43 (89.79, 92.83)
Attitude towards HIV/AIDS (Express accepting attitude on all four indicators, *n* = 2954)							
Yes	557	32.3 (28.6, 36.23)	339	27.45 (23.16, 32.21)		895	30.28 (27.4, 33.27)

Attitude towards HIV/AIDS is the combination of those that say ‘Yes’ in all these four indicators, namely (1) willingness to care for a relative with HIV/AIDS in their own home, (2) would they buy vegetables from a shopkeeper or vendor who has HIV/AIDS, (3) female teacher who has HIV/AIDS but is not sick should be allowed to continue teaching and, (4) would not want to keep a secret that a family member got infected with HIV/AIDS. Prevention towards HIV/AIDS is the combination of those that say ‘Yes’ to (a) have one sex partner only, who has no other partners, and (b) always use condoms and one sex partner only, who has no other partners. PMTCT is the combination of those that indicated ‘YES’ to the following questions on those who know that HIV/AIDS can be transmitted from mother to her baby (a) during pregnancy, (b) during delivery, and (c) by breastfeeding.

**Table 3:** Logistic analysis of respondent’s knowledge, attitudes, and prevention towards HIV/AIDS by individual-, household socioeconomic- and community-level characteristics.

Characteristic	Knowledge of HIV/AIDS		Prevention towards HIV/AIDS		Prevention of Transmission from Mother to Child (PMTCT)		Attitude towards HIV/AIDS	
	OR (95%CI)	*p*	OR (95%CI)	*p*	OR (95%CI)	*p*	OR (95%CI)	*p*
***Individual-level factors***								
**Gender**								
Male	Reference		Reference		Reference		Reference	
Female	0.88 (0.41, 1.87)	0.741	1.19 (0.72, 1.98)	0.482	2.83 (2, 3.99)	<0.001	1.26 (0.89, 1.77)	0.184
**Respondents Age**								
15–19	Reference		Reference		Reference		Reference	
20–24	5 (1.19, 21.53)	0.028	0.8 (0.39, 1.66)	0.566	1.46 (0.85, 2.52)	0.169	0.86 (0.51, 1.46)	0.597
25–29	4.2 (1.03,17.10)	0.045	0.84 (0.38, 1.85)	0.676	1.59 (0.89, 2.84)	0.111	0.98 (0.58, 1.66)	0.953
30–34	1.47 (0.35, 6.19)	0.592	0.73 (0.33, 1.61)	0.438	0.95 (0.51, 1.77)	0.891	1.52 (0.86, 2.68)	0.147
35–39	1.91 (0.45, 8.12)	0.378	0.85 (0.35, 2.03)	0.720	1.73 (0.93, 3.19)	0.079	0.75 (0.41, 1.39)	0.373
40–44	4.24 (0.74, 24.16)	0.103	1.17 (0.38, 3.57)	0.770	1.79 (0.87, 3.68)	0.113	0.51 (0.26, 1.03)	0.062
45–49	4.23 (0.92, 19.29)	0.062	1.12 (0.42, 3.01)	0.812	1.36 (0.69, 2.67)	0.366	0.68 (0.35, 1.33)	0.267
**Marital Status**								
Never married	Reference		Reference		Reference		Reference	
Currently married	0.38 (0.13, 1.11)	0.078	0.76 (0.45, 1.28)	0.314	1.12 (0.75, 1.67)	0.562	0.79 (0.53, 1.18)	0.267
Formerly married	0.49 (0.08, 2.85)	0.430	0.9 (0.35, 2.3)	0.829	1.13 (0.59, 2.13)	0.705	0.64 (0.34, 1.18)	0.156
**Educational Status**								
No education	Reference		Reference		Reference		Reference	
Primary	3.21 (1.49, 6.9)	0.003	0.58 (0.22, 1.53)	0.279	0.98 (0.4, 2.43)	0.982	0.59 (0.23, 1.47)	0.259
Secondary or more	2.28 (0.41, 12.57)	0.342	0.79 (0.24, 2.57)	0.706	0.95 (0.34, 2.62)	0.923	0.95 (0.34, 2.63)	0.923
**Working Status**								
Not working	Reference		Reference		Reference		Reference	
Working	0.76 (0.37, 1.56)	0.460	1.28 (0.75, 2.2)	0.359	0.81 (0.56, 1.16)	0.259	0.88 (0.62, 1.25)	0.495
**Religion**								
Christianity	Reference		Reference		Reference		Reference	
Others ^$^	0.26 (0.12, 0.55)	<0.001	0.25 (0.11, 0.54)	<0.001	0.8 (0.37, 1.75)	0.591	0.58 (0.29, 1.17)	0.134
**Migration Status**								
Yes	Reference		Reference		Reference		Reference	
No	1.12 (0.26, 4.71)	0.873	1.61 (0.85, 3.06)	0.140	0.99 (0.63, 1.55)	0.972	1.62 (0.97, 2.71)	0.061
***Household-level factors***								
**Household Wealth Index**								
Poor	Reference		Reference		Reference		Reference	
Middle	10.03 (2.06, 48.83)	0.004	1.5 (0.89, 2.5)	0.120	1.05 (0.73, 1.15)	0.776	1.06 (0.76, 1.48)	0.711
Rich	5.78 (0.7, 47.53)	0.103	1.32 (0.75, 2.32)	0.331	0.84 (0.57, 1.25)	0.415	1.76 (1.23, 2.51)	0.002
**Literacy**								
Can read whole sentence	Reference		Reference		Reference		Reference	
Cannot read whole sentence ^#^	0.06 (0.01, 0.29)	<0.001	1.08 (0.5, 2.32)	0.830	1 (0.46, 2.2)	0.980	1.23 (0.63, 2.39)	0.528
**Regular Media Exposure**								
No	Reference		Reference		Reference		Reference	
Yes	3.68 (1.44, 9.4)	0.006	2.7 (1.14, 6.39)	0.023	1.14 (0.37, 3.45)	0.811	5.65 (1.2, 26.4)	0.028
***Community-level factors***								
**Area of Residence**								
Rural	Reference		Reference		Reference		Reference	
Urban	15.37 (2.33, 101.39)	0.005	0.58 (0.39, 0.87)	0.009	0.80 (0.60, 1.07)	0.148	0.99 (0.76, 1.32)	0.964
**Caste/Tribe**								
Scheduled Tribe	Reference		Reference		Reference		Reference	
Other Backward Class	0.2 (0.05, 0.69)	0.011	10.9 (1.94, 61.64)	0.007	0.35 (0.14, 0.86)	0.022	0.19 (0.07, 0.56)	0.002

^$^ Hindu, Muslim, Buddhist/neo-Buddhist, and other religion; ^#^ cannot read at all, can read only parts of a sentence. Attitude towards HIV/AIDS is the combination of those that say ‘Yes’ in all these four indicators, namely: (1) willingness to care for a relative with HIV/AIDS in their own home, (2) would they buy vegetables from a shopkeeper or vendor who has HIV/AIDS, (3) female teacher who has HIV/AIDS but is not sick should be allowed to continue teaching and, (4) would not want to keep a secret that a family member got infected with HIV/AIDS. Prevention towards HIV/AIDS is the combination of those that say ‘Yes’ to (a) have one sex partner only, who has no other partners, and (b) always use condoms and one sex partner only, who has no other partners. PMTCT is the combination of those that indicated ‘YES’ to the following questions on those who know that HIV/AIDS can be transmitted from mother to her baby (a) during pregnancy, (b) during delivery, and (c) by breastfeeding.

**Table 4:** Adjusted Odds ratios (95% confidence intervals) for respondents’ knowledge, attitudes, and prevention towards HIV/AIDS: a multivariate analysis.

Characteristic	Knowledge of HIV/AIDS		Prevention towards HIV/AIDS		Prevention of Transmission from Mother to Child (PMTCT)		Attitude towards HIV/AIDS	
	AOR (95%CI)	*p*	AOR (95%CI)	*p*	AOR (95%CI)	*p*	AOR (95%CI)	*p*
***Individual-level factors***								
**Gender**								
Male	N.S	N.S	N.S	N.S	Reference		N.S	N.S
Female	N.S	N.S	N.S	N.S	3.12 (2.34, 4.16)	<0.001	N.S	N.S
**Respondents Age**								
15–19	N.S	N.S	N.S	N.S	Reference		N.S	N.S
20–24	N.S	N.S	N.S	N.S	1.39 (0.82, 2.34)	0.210	N.S	N.S
25–29	N.S	N.S	N.S	N.S	1.57 (0.95, 2.59)	0.073	N.S	N.S
30–34					0.95 (0.57, 1.59)	0.870		
35–39	N.S	N.S	N.S	N.S	1.74 (1.05, 2.87)	0.030	N.S	N.S
40–44	N.S	N.S	N.S	N.S	1.80 (0.97, 3.35)	0.060	N.S	N.S
45–49	N.S	N.S	N.S	N.S	1.40 (0.82, 2.4)	0.208	N.S	N.S
**Religion**								
Christianity	Reference		Reference		N.S		N.S	N.S
Others ^$^	0.28 (0.14, 0.55)	<0.001	0.28 (0.12, 0.64)	0.002	N.S		N.S	N.S
**Educational Status**								
No education	Reference		N.S	N.S	N.S	N.S	N.S	N.S
Primary	3.05 (1.46, 6.37)	0.003	N.S	N.S	N.S	N.S	N.S	N.S
Secondary or more	2.54 (0.5, 12.87)	0.259	N.S	N.S	N.S	N.S	N.S	N.S
**Caste/Tribe**								
Scheduled Tribe	Reference		Reference		Reference		Reference	
Other Backward Class	0.21 (0.06, 0.73)	0.014	8.60 (1.72, 43.02)	0.009	0.34 (0.14, 0.82)	0.017	0.18 (0.07, 0.44)	<0.001
***Household-level factors***								
**Household Wealth Index**								
Poor	Reference		N.S	N.S	N.S	N.S	Reference	
Middle	10.64 (2.34, 48.24)	0.002	N.S	N.S	N.S	N.S	1.15 (0.82, 1.6)	0.404
Rich	5.00 (0.53, 47.22)	0.159	N.S	N.S	N.S	N.S	1.86 (1.35, 2.56)	<0.001
**Literacy**								
Who can read whole sentence	Reference		N.S	N.S	N.S	N.S	N.S	N.S
Can not read whole sentence ^#^	0.07 (0.01, 0.31)	<0.001	N.S	N.S	N.S	N.S	N.S	N.S
**Regular Media Exposure**								
No	Reference		Reference		N.S	N.S	Reference	
Yes	2.75 (1.22, 6.17)	0.014	2.84 (1.36, 5.95)	0.005	N.S	N.S	5.34 (1.3, 21.82)	0.02
***Community-level factors***								
**Area of Residence**								
Rural	Reference		N.S	N.S	N.S	N.S	N.S	N.S
Urban	14.4 (2.67, 78.54)	0.002	N.S	N.S	N.S	N.S	N.S	N.S

^$^ Hindu, Muslim, Buddhist/neo-Buddhist, and other religion; ^#^ cannot read at all, can read only parts of a sentence. Attitude towards HIV/AIDS is the combination of those that say ‘Yes’ in all these four indicators, namely: (1) willingness to care for a relative with HIV/AIDS in their own home, (2) would they buy vegetables from a shopkeeper or vendor who has HIV/AIDS, (3) female teacher who has HIV/AIDS but is not sick should be allowed to continue teaching and, (4) would not want to keep a secret that a family member got infected with HIV/AIDS. Prevention towards HIV/AIDS is the combination of those that say ‘Yes’ to (a) have one sex partner only, who has no other partners, and (b) always use condoms and one sex partner only, who has no other partners. PMTCT is the combination of those that indicated ‘YES’ to the following questions on those who know that HIV/AIDS can be transmitted from mother to her baby (a) during pregnancy, (b) during delivery, and (c) by breastfeeding.

## Data Availability

The study was based on an analysis of existing survey datasets that are available to apply for online, with all identifier information removed. Written informed consent for the present analysis was not necessary because secondary data analysis did not involve interaction with the participants. The data collection methods for the NFHS-4 data used in this analysis, including the consent process, have been previously described [12]. Written informed consent for the present analysis was not necessary because secondary data analysis did not involve interaction with the participants.

## Supplementary Materials

The following supporting information can be downloaded at: https://www.mdpi.com/article/10.3390/ijerph19010440/s1, Table S1 for details.
