# Supplementary material for: Factors Associated with Knowledge, Attitudes, and Prevention towards HIV/AIDS among Adults 15–49 Years in Mizoram, North East India: A Cross-Sectional Study
Source: Int J Environ Res Public Health. 2021 Dec 31;19(1):440. doi: 10.3390/ijerph19010440 (PMC8744648; doi:10.3390/ijerph19010440)
Supplement: Supplementary file 1 [file ijerph-19-00440-s001.zip › ijerph-1445203-SI.pdf]

Supplementary Table S1

Indicators	Knowledge of HIV/AIDS	Prevention towards HIV/AIDS	Prevention of transmission from mother to child (PMTCT)	Attitude towards HIV/AIDS
	**% (95%CI)**	**% (95%CI)**	**% (95%CI)**	**% (95%CI)**
Individual-level factors				
**Gender**				
Male	98.5 (97.7 ,99)	84.3 (80.8, 87.2)***	33.3 (29.6, 37.1)	21.3 (17.9, 25.3)***
Female	97.6 (96.8 ,98.3)	82.8 (79.9, 85.3)	54.5 (50.7, 58.3)	28.3 (25, 31.9)
**Age Group**				
15-19	98.8 (97.5 ,99.4)	82.6 (77.1, 86.9)	37.1 (30.8, 43.9)**	29.3 (23.3, 36)*
20-24	98.9 (97.4 ,99.5)	83.3 (77.3, 87.9)	43.8 (36.5, 51.3)	23.9 (18.1, 30.9)
25-29	98.2 (96.6 ,99.1)	82.9 (77.3, 87.3)	48.2 (41.5, 54.9)	26.3 (20.7, 32.8)
30-34	96.9 (94.9 ,98.1)	84.2 (78.3, 88.7)	42.5 (35.7, 49.5)	34.9 (27.8, 42.8)
35-39	97.1 (95 ,98.3)	81 (74.3, 86.3)	53.6 (46.5, 60.6)	20.7 (15.6, 27)
40-44	98.3 (95.8 ,99.4)	85.9 (77.9, 91.4)	60 (50, 69.4)	16.9 (11.6, 23.8)
45-49	98.1 (96.3 ,99.1)	85.7 (79.1, 90.4)	37 (30.1, 44.5)	18.8 (13.5, 25.4)
**Marital Status**				
Never married	99.3 (98.6 ,99.6)***	84.2 (80.6, 87.2)	40.1 (35.7, 44.7)	28.9 (24.7, 33.4)
Currently married	96.9 (95.9 ,97.6)	82.3 (79.3, 85)	48.2 (44.5, 51.9)	22.7 (19.7, 26)
Formerly married	99.1 (97.2 ,99.7)	87.1 (78.3, 92.6)	48.9 (38.2, 59.7)	22.7 (14.9, 33)
**Educational Status**				
No education	68.2 (58.3 ,76.8)***	45.7 (35, 56.9)***	26.9 (18.2, 37.8)***	13.7 (7.7, 23.4)***
Primary	95.6 (92.9 ,97.3)	76.1 (70.3, 81.1)	46.2 (39.7, 52.8)	14.8 (11, 19.6)
Secondary	99.7 (99.4 ,99.9)	86.2 (83.8, 88.2)	45.7 (42.6, 48.8)	27.1 (24.3, 30.1)
**Working Status**				
Not working	98 (97.1 ,98.6)	82.6 (79.2, 85.5)	51.3 (46.9, 55.6)***	28.3 (24.5, 32.4)
Working	98.1 (97.3 ,98.6)	84.2 (81.3, 86.8)	40 (36.5, 43.5)	22.7 (19.6, 26.1)
**Religion**				
Christianity	98.6 (98.1 ,99)***	84.6 (82.4, 86.6)***	46.1 (43.2, 49)***	26 (23.5, 28.7)***
Other religion	86.5 (79.8 ,91.2)	60.3 (49.3, 70.2)	24.3 (16.6, 34)	9.5 (5.6, 15.7)
**Migration Status**				
Yes	99.3 (97.8 ,99.8)*	83.4 (75.2, 89.2)*	39.7 (31.5, 48.6)	18.9 (12.8, 27)
No	97.9 (97.2 ,98.3)	83.5 (81.2, 85.5)	45.7 (42.8, 48.7)	26 (23.5, 28.8)
Household-level factors				
**Household Wealth Index**				
Poor	93.0 (91.0 ,94.6)***	75.9 (72.3, 79.2)***	42.2 (38, 46.4)***	19.3 (16.2, 22.9)***
Middle	99.8 (99.4 ,100)	88 (84.6, 90.8)	47.2 (42.4, 52.1)	21.8 (18.1, 26.1)
Rich	99.9 (99.4 ,100)	85 (81, 88.3)	45.3 (40.5, 50.2)	31 (26.7, 35.8)
**Regular Media Exposure**				
Yes	98.6 (98.1 ,98.9)***	84.1 (82, 86.1)***	26.9 (15.2, 43.2)***	25.6 (23.1, 28.2)***
No	69.2 (55.5 ,80.3)	46.2 (32.7, 60.4)	45.4 (42.5, 48.2)	3.6 (0.9, 12.8)
**Literacy**				
Can read whole sentence	99.7 (99.4 ,99.8)***	85.7 (83.4, 87.7) ***	45.7 (42.7, 48.7)***	26.2 (23.6, 29)***
Can't read whole sentence	79.9 (74.4 ,84.4)	58.6 (51.7, 65.2)	37.9 (31.6, 44.7)	14.4 (10.4, 19.6)
Community-level factors				
**Area of Residence**				

Rural	95 (93.6 ,96.1)***	80.3 (77.5, 82.9)***	43.2 (39.8, 46.6)**	21.2 (18.5, 24.1)*
Urban	99.9 (99.7 ,100)	85.4 (82.2, 88)	46.1 (42.2, 50.2)	27.6 (24.1, 31.4)
**Caste/Tribe**				
Scheduled Tribe	98.1 (97.6 ,98.6)*	83.7 (81.5, 85.7)**	45.7 (42.8, 48.5)*	25.6 (23.2, 28.3)**
Other backward class	94.2 (84.9 ,97.9)	72.9 (50.4, 87.7)	19 (10.9, 31)	6.2 (2.9, 12.5)

Significance (p value) = * < 0.05, ** < 0.001, *** < 0.0001, $Hindu, Muslim, Buddhist/neo-Buddhist, and other religion; #cannot read at all, read only parts of sentence. Attitude towards HIV/AIDS is the combination of those that say 'Yes' in all these 4 indicators namely: (1) willingness to care for a relative with HIV/AIDS in own home, (2) would they buy vegetables from a shopkeeper or vendor who has HIV/AIDS, (3) female teacher who has HIV/AIDS but is not sick should be allowed to continue teaching and, (4) would not want to keep a secret that a family member got infected with HIV/AIDS.. Prevention towards HIV/AIDS is the combination of those that say 'Yes' to (a) have 1 sex partner only, who has no other partners and (b) always use condoms and 1 sex partner only, who has no other partners. PTMT is the combination of those that indicated 'YES' to the following questions on those who know that HIV/AIDS can be transmitted from mother to her baby (a) during pregnancy, (b) during delivery and, (c) by breastfeeding.
